# Gait Planning and Load-Bearing Capacity Analysis of Bionic Quadrupedal Robot Actuated by Water Hydraulic Artificial Muscles

**DOI:** 10.3390/biomimetics11010024

**Published:** 2026-01-01

**Authors:** Jun Li, Zengmeng Zhang, Shoujie Feng, Yong Yang, Yongjun Gong

**Affiliations:** 1 Naval Architecture and Ocean Engineering College, Dalian Maritime University, Dalian 116026, China; lj15865379796@dlmu.edu.cn (J.L.); p2279654041@163.com (S.F.); 17615103064@163.com (Y.Y.); gyj@dlmu.edu.cn (Y.G.); 2 Key Laboratory of Rescue and Salvage Engineering Liaoning Province, Dalian 116026, China

**Keywords:** quadrupedal robot, load analysis, water hydraulic artificial muscles, gait planning

## Abstract

The gecko-inspired crawling robot driven by water hydraulic artificial muscles (WHAMs) incorporates the stable structural characteristics of geckos, making it particularly suitable for operation in aquatic environments. Conventional crawling robots typically employ electric or oil hydraulic actuation systems, which require complex sealing and waterproof designs when working in water. This study presented a bionic quadruped robot actuated by WHAMs that fundamentally circumvents waterproofing challenges. Although the joint module can dynamically adjust its output torque according to requirements, there has been a lack of theoretical basis for load adjustment. This research established the relationship between the leg joint load and the WHAM pressure difference, resulting in a pressure difference–load model for the leg joint. Through gait planning analysis, the maximum supporting force during robot motion was determined. Experimental tests on a single-leg prototype demonstrated a maximum static load capacity of 23 kg under stationary conditions, while during cycloidal motion the dynamic load capacity reached 10 kg. Both values satisfied the supporting force requirements of the planned gait. Furthermore, the pressure difference–load model showed good agreement with experimental results, providing theoretical guidance for load adjustment in leg joints.

## 1. Introduction

The classification of artificial muscles includes pneumatic artificial muscles [1,2,3,4], hydraulic artificial muscles [5,6], electroactive artificial muscles [7], etc., which have their own unique advantages in different application fields. The water medium has a wide range of sources and clean characteristics, and water hydraulic artificial muscle (WHAM) has attracted the interest of some researchers. Regarding principal research, Jia et al. [8] proposed a biomimetic robot flexible module supported by springs actuated by WHAM. A static model of the flexible module was established, and the deviation between the results and the model was experimentally discussed, thereby laying the foundation for the design and manufacturing of integrated flexible robots. Zhang et al. [9] conducted an analysis and study on the failure mechanism of high-strength WHAM by designing an orthogonal experiment with four factors and two levels. Based on the orthogonal analysis, the structural parameters of high-strength WHAM were obtained, which improved the application reliability of WHAM. In the field of robotics, researchers have attempted to apply artificial muscles to robots and have achieved good results. Feng et al. [10] used 29 hydraulic artificial muscles to manufacture a 7-degree-of-freedom soft robotic arm with a span of over 1.5 m and conducted performance tests. The results indicated that the robot had strong impact resistance. Yang et al. [11] used hydraulic artificial muscles to drive multistage underwater robotic arms, optimized the static mathematical model of hydraulic artificial muscles, established forward and inverse kinematic models, and verified the effectiveness of model solving through experiments. Ito et al. [12] proposed a pipeline wire installation assistance robot using artificial muscles that can reduce the workload of wire installation and has practical application value.

**Figure 1:**
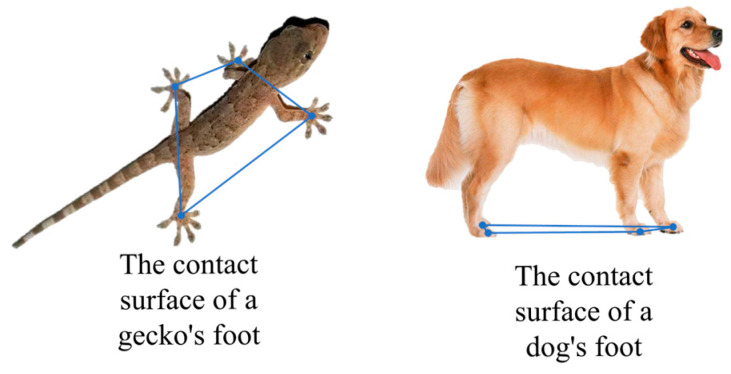
Arrangement of animal legs.

In animals, leg joints are distributed in different configurations, being laterally positioned in geckos and crocodiles [13,14] and underneath positioned in horses [15] and dogs [16,17], as shown in Figure 1. Horses possess legs arranged underneath their body, providing a strong load-bearing capacity with low resistance [18]. However, the ground contact area of their feet is relatively small compared to their body area. Drawing inspiration from streamlined structures and load-bearing requirements, extensive research has been conducted on both hydraulic and electric actuation systems. For gecko-inspired robots [19,20,21,22], the leg joints are distributed on both sides of the body, resulting in a relatively larger ground contact area than the body area and a lower body height. This configuration maintains stability even when the center of mass shifts, thereby reducing the control demands on the body.

The load capacity of a robot is an important performance indicator. Researchers have conducted several exploratory studies on the load-bearing capacities of robots. Kim et al. [23] conducted research on passenger bipedal robots, analyzed passenger weight data, and improved the balance control method, resulting in an improved performance and stability of the robot. Deng et al. [24] analyzed the use of redundant legs in hexapod robots for object handling, introducing practical gaits for single and double leg handling, and solving problems in motion planning, such as how to estimate the mass of an object and how to calculate joint motion based on the trajectory of the object’s center of gravity. In addition, research on quadrupedal robots is more extensive, with in-depth studies on gait design [25,26,27], control [28,29], and other areas. Regarding the load aspect of quadruped robots, Gu et al. [30] proposed an adaptive control method for quadruped robots that targets unknown loads and enables human–machine interaction. The method identifies the actual center-of-mass position of the torso, estimates the actual center-of-mass position of the robot through the torque generated by pulses, and achieves stability control for carrying unknown loads on quadrupedal robots. Improved the carrying capacity and human–machine interaction ability of quadrupedal robots, reducing the impact of external interference. Hydraulic robots have received widespread attention owing to their higher load-bearing capacity, but numerous components and high-pressure pipes present challenges to robot design and layout. Zong et al. [31] analyzed the load capacity of a hydraulic quadrupedal robot using lightweight and modular design and found that it has good motion capability while ensuring load capacity.

At present, there is a great deal of research on quadruped robots with motors as the driving module, and research on quadruped robots actuated by WHAMs is still in its infancy. WHAMs can generate a large output force, and their small size and high force-to-weight ratio make them suitable for shallow-water operations. The quadrupedal robot actuated by WHAMs provides a new idea for underwater crawling robots. This study analyzes the load capacity of robots actuated by WHAMs, explores the maximum load capacity of leg joints, and provides a theoretical basis for adjusting leg joint loads, thereby providing a theoretical reference for subsequent underwater operations.

## 2. Robot Structure

### 2.1. Design and Analysis of Joint Module

**Figure 2:**
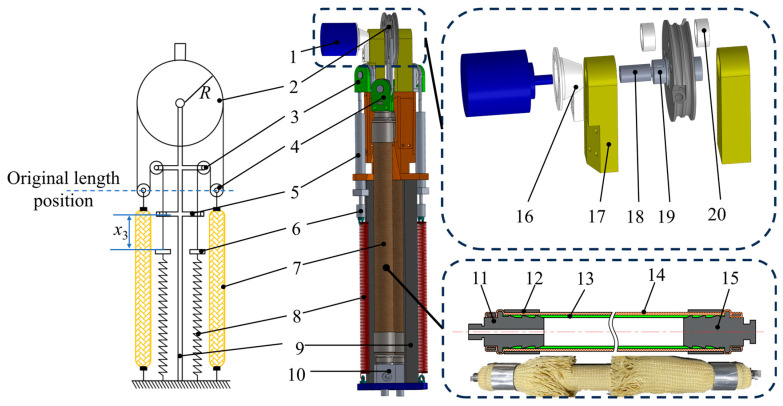
Joint module: 1. Waterproof angle sensor, 2. Joint wheel, 3. Fixed pulley, 4. Movable pulley, 5. Limiting bolt, 6. Limiting block, 7. WHAM, 8. Spring, 9. Support rod, 10. Water injection port, 11. Muscle water injection connector, 12. Attachment ring, 13. Rubber tube, 14. Fiber woven mesh, 15. Closed connector, 16. Sensor fixing component, 17. Joint wheel support, 18. Sensor connector, 19. Joint shaft, 20. Bushing.

The exterior of WHAM is constructed from high-strength fiber materials, while the interior is lined with rubber tubes. This design ensures good contraction characteristics while maintaining sufficient reliability. Its structure is illustrated in Figure 2.

**Figure 3:**
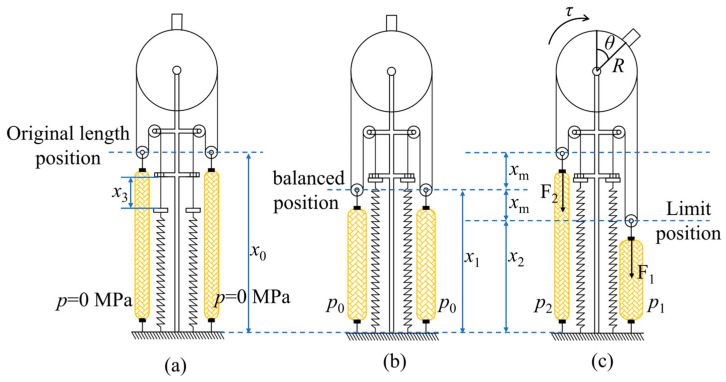
The working principle of the joint module. (**a**) Operating state of the joint module when the WHAMs are unpressurized; (**b**) Operating state of the joint module when the pressures in both WHAMs are balanced; (**c**) Operating state of the joint module when the pressure in one WHAM increases while the pressure in the other decreases.

WHAMs can only perform stretching and contraction. We converted the stretching and contraction movements of the WHAMs into rotational movements. By taking advantage of the antagonistic effect of the two WHAMs, the linear motion of the WHAMs was converted into rotational motion. A pulley block structure was adopted to increase the rotation angle range, and a joint module was designed. The structure is illustrated in Figure 2. The working principle of the joint module is shown in Figure 3.

In the joint module, one end of the steel wire rope is fixed to the joint wheel, while the other end is secured to a limiting block. A spring maintains tension on the steel wire rope when the WHAM is at its initial length, thereby preventing the rope from derailing from the pulley. The Limiting bolt allows for adjustment of the distance relative to the limiting block. This distance defines the pre-adjusted length of the steel wire rope in the joint module, which directly determines the output torque of the joint module. The output force of the WHAM is transmitted to the steel wire rope via a movable pulley mechanism. The joint wheel is rigidly connected to the joint shaft through a key joint. An angle sensor, attached to the joint shaft via a sensor connector, collects the rotation angle signal of the joint wheel. The joint wheel achieves different rotation angles when the two WHAMs are pressurized to different levels. However, the movable pulley mechanism reduces the output force transmitted through the steel wire rope to half the original value while simultaneously amplifying the contraction displacement of the WHAMs, thereby extending the range of joint rotation. When both WHAMs operate at a balanced position with balanced pressures, the forces on either side of the joint wheel equalize. Consequently, the joint wheel remained stationary in the neutral position. *x*_0_ denotes the initial length of the WHAM in the joint module, *x*_1_ represents the length when the pressure of two WHAMs is balanced. *x*_2_ indicates the length of the WHAM at maximum contraction, and *x*_3_ represents the pre-adjusted length of the steel wire rope. The rotation angle of the joint pulley is represented by *θ*, and its radius by *R*. The tensile force in the WHAM in the relaxed state is denoted as *F*_1_, and that in the contracted state as *F*_2_. When the WHAMs are unpressurized, the WHAMs remain at its initial length. Upon pressurization until equal pressure is achieved in both antagonistic muscles, the WHAMs reach its balanced position. When the pressure in one WHAM increases, causing it to contract by a distance *x*_m_, the pressure in the opposing WHAM decreases, resulting in its elongation by an equal distance. The contracting WHAM drives the joint wheel to rotate in the direction of contraction, thereby enabling the conversion from the pressure differential to joint rotation.

The driving characteristics [32] of WHAMs are as follows:

(1) $$F_{m}\left(\varepsilon ,p\right)=\kappa_{F}(\pi r_{0}^{2})p\left[a_{m}{\left(1-\kappa_{\varepsilon }\varepsilon \right)}^{2}-b_{m}\right],$$

where $F_{m}\left(\varepsilon ,p\right)$ represents the output force of the WHAM. p represents the pressure of the WHAM. $r_{0}$ represents the initial radius of the WHAM and ε represents the contraction rate of the WHAM. ε is given by:

(2) $$\varepsilon =\frac{l_{0}-l}{l_{0}},$$

where $l_{0}$ and l represent the initial and current lengths of the WHAM, respectively. $a_{m}$ and $b_{m}$ represent the parameters related to the initial weaving angle. $a_{m}$ and $b_{m}$ are given by:

(3) $$a_{m}=\frac{3}{\tan^{2}\alpha_{0}},\;b_{m}=\frac{1}{\sin^{2}\alpha_{0}},$$

where $\alpha_{0}$ represents the initial weaving angle. $\kappa_{F}$ and $\kappa_{\varepsilon }$ represent the stress and strain adjustment parameters, respectively. $\kappa_{F}$ and $\kappa_{\varepsilon }$ are given by:

(4) $$\kappa_{\varepsilon }=\frac{1-\sqrt{b_{m}/a_{m}}}{\varepsilon_{\max}};\;\kappa_{F}=\frac{F_{\max}}{\left(\pi r_{0}^{2})p(a_{m}-b_{m}\right)},$$

$\varepsilon_{\max}$ and $F_{\max}$ represent the maximum initial contraction rate and maximum output force of the WHAM. Considering the changes in the contraction rate of WHAM when it is filled with water and the pressure of the filling fluid, the contraction rate and pressure of the contracting WHAM are $\varepsilon_{1}=\varepsilon_{0}+R\theta /(2l_{0})$, $p_{1}=p_{0}+\Delta p/2$, and the contraction rate and pressure of the relaxing WHAM are $\varepsilon_{2}=\varepsilon_{0}-R\theta /(2l_{0})$, $p_{2}=p_{0}-\Delta p/2$. $\varepsilon_{0}$ represents the initial shrinkage rate, $p_{0}$ represents the initial internal pressure of the WHAMs, Δp represents the pressure difference between the two WHAMs, θ represents the rotation angle of the joint wheel, and *R* represents the radius of the joint wheel. Obtain the tensile force $F_{1}$ when WHAM contracts and the tensile force $F_{2}$ when it relaxes.

(5) $$\left\{\begin{array}{l} F_{1}=\kappa_{F}\left(\pi r_{0}^{2}\right)\left(p_{0}+\frac{\Delta p}{2}\right)\left[a_{m}{\left(1-\kappa_{\varepsilon }\varepsilon_{0}-\kappa_{\varepsilon }\frac{R\theta }{2l_{0}}\right)}^{2}-b_{m}\right]\\ F_{2}=\kappa_{F}\left(\pi r_{0}^{2}\right)\left(p_{0}-\frac{\Delta p}{2}\right)\left[a_{m}{\left(1-\kappa_{\varepsilon }\varepsilon_{0}+\kappa_{\varepsilon }\frac{R\theta }{2l_{0}}\right)}^{2}-b_{m}\right] \end{array}\right.,$$

Two hydraulic artificial muscles act against each other on the joint wheel, and half the difference in tension between the two is multiplied by the radius of the joint wheel to obtain the output torque of the joint wheel. The output torque of the joint wheel is

(6) $$\tau =\frac{R}{2}\left(F_{1}-F_{2}\right)=\frac{R}{2}\left(\left[k_{1}+k_{3}{\left(R\theta \right)}^{2}\right]\Delta p-k_{2}p_{0}R\theta \right),$$

$k_{1}$, $k_{2}$ and $k_{3}$ are given by:

(7) $$k_{1}=\kappa_{F}(\pi r_{0}^{2})\left[a_{m}{\left(1-\kappa_{\varepsilon }\varepsilon_{0}\right)}^{2}-b_{m}\right],$$

(8) $$k_{2}=2\kappa_{F}(\pi r_{0}^{2})\left[a_{m}\left(1-\kappa_{\varepsilon }\varepsilon_{0}\right)\frac{\kappa_{\varepsilon }}{l_{0}}\right],$$

(9) $$k_{3}=\kappa_{F}(\pi r_{0}^{2})\frac{a_{m}{\kappa_{\varepsilon }}^{2}}{4{l_{0}}^{2}},$$

According to Equation (6), the relationship between the pressure difference of WHAM and the rotation angle and torque is further obtained as:

(10) $$\Delta p=\frac{k_{2}p_{0}R\theta }{k_{1}+k_{3}{\left(R\theta \right)}^{2}}+\frac{2\tau }{k_{1}R+k_{3}R{\left(R\theta \right)}^{2}},$$

The pre-adjusted length of the steel wire ropes determines the initial contraction rate of WHAM. By adjusting the position of the limit bolt, the pre-adjusted length measurement $x_{3}$ of the steel wire rope can be adjusted, thereby enabling the dynamic adjustment of the output torque and rotation angle range of the module. The relationship between the pre-adjusted length of the steel wire ropes and the initial contraction rate of WHAM is as follows:

(11) $$\varepsilon_{0}=\frac{x_{3}}{2l_{0}},$$

The parameters of the joint module are listed in Table 1.

**Figure 4:**
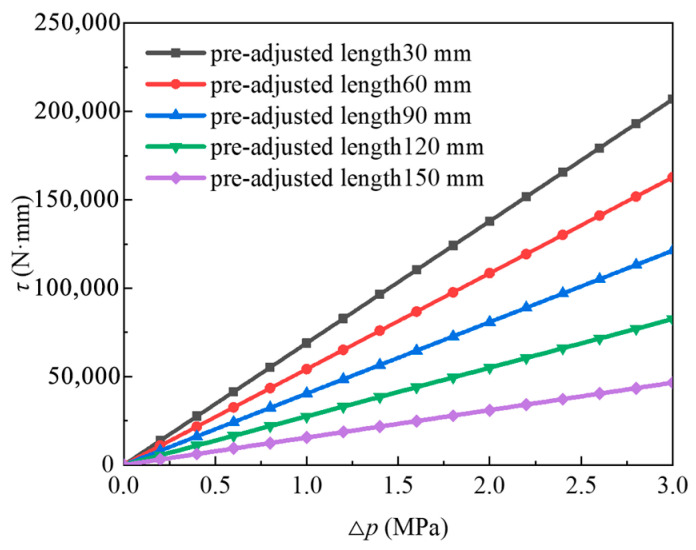
The relationship between the output torque of the joint module and the pressure difference between the two WHAMs.

When the rotation angle of the joint wheel was 0°, the output torque of the joint module was at its maximum. By substituting the parameters in Table 1 into Equation (6), the variation in the output torque of the joint module with the pressure difference between the two WHAMs under different pre-adjusted lengths of the steel wire ropes is shown in Figure 4.

The output torque of the joint wheel increases as the pressure difference between the two WHAMs increases. The smaller the pre-adjusted length of the steel wire rope, the greater the output torque will be under the same pressure difference, but it is not the case that the smaller the pre-adjusted length, the better. If the pre-adjustment length is too small, it leads to a reduction in the angle range of the joint wheel, resulting in incomplete movement. Therefore, in actual situations, the pre-adjusted length of the steel wire rope should be reasonably adjusted to maximize the output torque while satisfying the range of motion.

The joint module realizes the conversion of the telescopic movement of WHAM to rotational movement. Meanwhile, it can adjust the pre-adjusted length of the steel wire rope to adapt to different load conditions. Based on its working principle, it can be used in engineering applications.

### 2.2. Structural Design of Quadruped Robot Actuated by WHAMs

**Figure 5:**
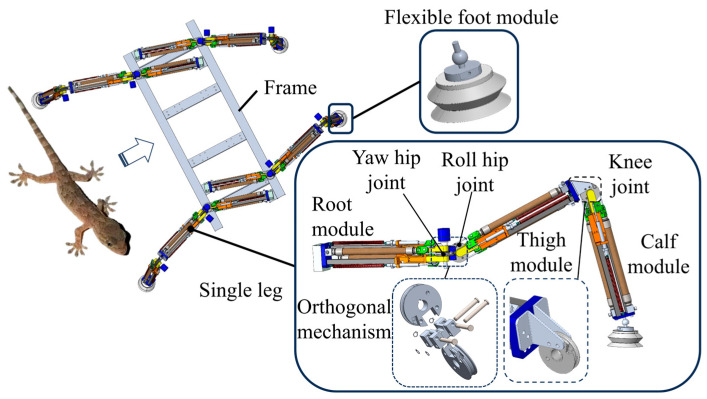
Water hydraulic quadrupedal robot model.

The WHAM module converts the linear stretching motion of WHAM into the rotational motion of the joint wheel. Reference [33] provided a set of gecko parameters, with the specific parameters listed in Table 2. In this study, a WHAM-driven robot for underwater engineering operations was designed by imitating the side-mounted structure of geckos. In underwater environments, the interference of fuselage becomes more complex owing to the effects of waves and tides. Therefore, in underwater environments, the arrangement of legs on both sides has an advantage in terms of stability compared with the arrangement of legs at the bottom. Because the gecko’s leg joint has three degrees of freedom and the WHAM module has only one degree of freedom, a gecko-like leg joint structure was obtained by imitating the structural characteristics of the gecko’s leg joint and connecting three WHAM modules in series. Based on this, by imitating the body structure of geckos, a water-hydraulic quadrupedal robot actuated by artificial muscles and imitating geckos was obtained through a side-mounted design. The three-dimensional model is illustrated in Figure 5.

The overall structure of the robot includes a frame, leg joint, and flexible foot module. To reduce the weight of the robot, a frame was constructed with a groove structure made of aluminum alloy. The single leg is composed of three joint modules connected in series, serving as the root, thigh, and calf modules of the single leg. The root and thigh modules were connected by an orthogonal mechanism to form the yaw and roll hip joints. The thigh and calf modules were connected using a triangular plate to form the knee joint. To reduce the impact with the ground, a flexible module made of rubber is used at the foot end to reduce the impact strength with the ground.

## 3. Gait Planning and Torque Solving

### 3.1. Kinematic Analysis of the Leg Joint

**Figure 6:**
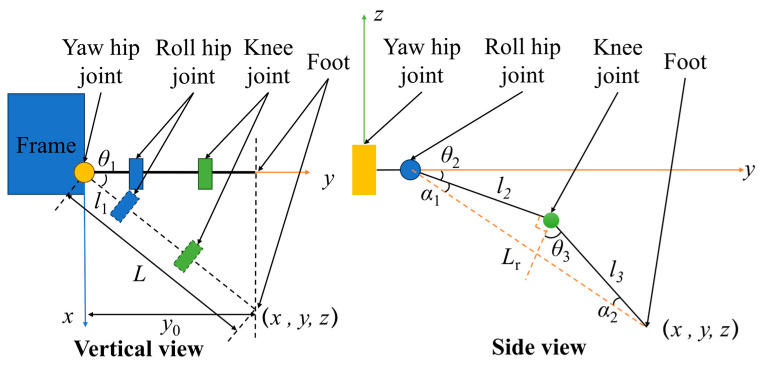
Inverse kinematic analysis of the leg joint.

Kinematic analysis is essential for achieving motion control of quadruped robots. Kinematic solutions are generally categorized into numerical methods and analytical methods. While numerical methods offer high versatility, they require extensive iterative computations and are better suited for joints with multiple degrees of freedom. Since the leg joint has only three degrees of freedom, employing the analytical method provides high computational efficiency and fast processing. Therefore, this study adopts the analytical method for solving leg joint kinematics. The leg joint structure is simplified, resulting in the kinematic analysis diagram shown in Figure 6.

*L* represents the distance from the foot end to the yaw hip joint in the vertical view of the quadruped robot. $l_{1}$ denotes the distance from the yaw hip joint to the roll hip joint. $l_{2}$ indicates the distance from the roll hip joint to the knee joint. $l_{3}$ represents the distance from the foot end to the knee joint. $L_{r}$ stands for the distance from the foot to the roll hip joint. The distance from the foot end to the rotation axis of the yaw hip joint is defined as *y*_0_. $\theta_{1}$, $\theta_{2}$, and $\theta_{3}$ represent the joint rotation angles. Two intermediate variables, $\alpha_{1}$ and $\alpha_{2}$, are defined, representing two internal angles of the triangle formed by the foot, roll hip joint, and knee joint. *y*_0_ is the distance from the foot end to the rotation axis of the yaw hip joint. First, based on the vertical view, the expression for $\theta_{1}$ is given by:

(12) $$\theta_{1}=\arctan(\frac{x}{y}),$$

The intermediate variable *L* is given by:

(13) $$L=\sqrt{x^{2}+y^{2}},$$

From the side view, the expressions for the intermediate variables $L_{r}$, $\alpha_{1}$, and $\alpha_{2}$ are given by:

(14) $$L_{r}=\sqrt{z^{2}+{\left(L-l_{1}\right)}^{2}},$$

(15) $$\alpha_{1}=arc\cos(\frac{{L_{r}}^{2}+{l_{2}}^{2}-{l_{3}}^{2}}{2l_{2}L_{r}}),$$

(16) $$\alpha_{2}=arc\cos(\frac{{L_{r}}^{2}-{l_{2}}^{2}+{l_{3}}^{2}}{2l_{3}L_{r}}),$$

Subsequently, the expressions for $\theta_{2}$ and $\theta_{3}$ are given by:

(17) $$\theta_{2}=\arctan(\frac{z}{L-l_{1}})+\alpha_{1},$$

(18) $$\theta_{3}=\frac{\pi }{2}-\alpha_{1}-\alpha_{2},$$

### 3.2. Structural Parameters and Supporting Force Analysis of the Quadruped Robot

**Figure 7:**
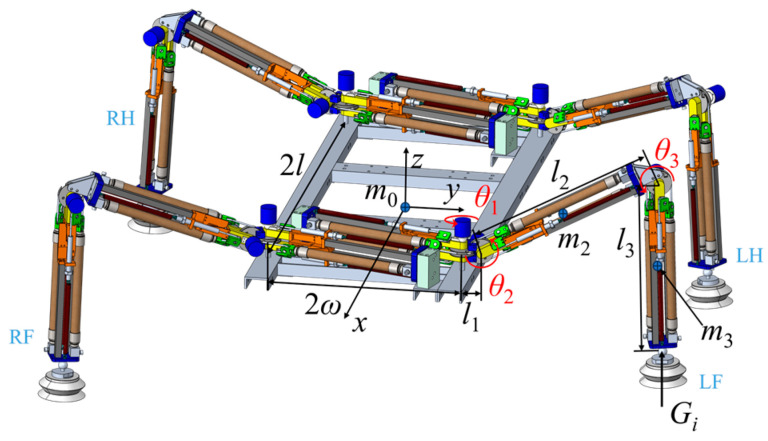
Supporting force analysis of the quadrupedal robot.

During the movement of the robot, the supporting force at the end of the foot reflects the load-bearing capacity of the leg joints of the quadrupedal robot. Therefore, a supporting force analysis was conducted on the quadrupedal robot, as shown in Figure 7.

LF is designated as the robot’s left front leg, LH as the left hind leg, RF as the right front leg, RH as the right hind leg. The supporting force at the end of each leg is denoted by *G_i_*, where *i* = 1, 2, 3, 4 correspond to LH, LF, RH, and RF, respectively. The distance between the yaw hip joints of the front and rear legs along the *x*-axis is represented by 2*l*, while the distance between the yaw hip joints of the left and right legs along the *y*-axis is represented by 2*ω*. The mass of the main body is denoted as $m_{0}$, the body weight is $G_{m}=m_{0}g$. *g* is the gravitational acceleration. The mass of the thigh module is denoted as $m_{2}$, and the mass of the calf module is denoted as $m_{3}$. The specific parameters of the quadrupedal robot are listed in Table 3. In Table 2, the thigh and calf lengths of the gecko are nearly equal, with a thigh-to-calf length ratio of 1.023. The quadrupedal bionic robot was designed to mimic the structural parameters of the gecko’s legs. Since the thigh module must ensure consistency in the length of the hydraulic artificial muscles and requires additional mechanisms to connect with the calf module, the final robot design features a thigh-to-calf length ratio of 1.122.

### 3.3. Gait Planning

**Figure 8:**
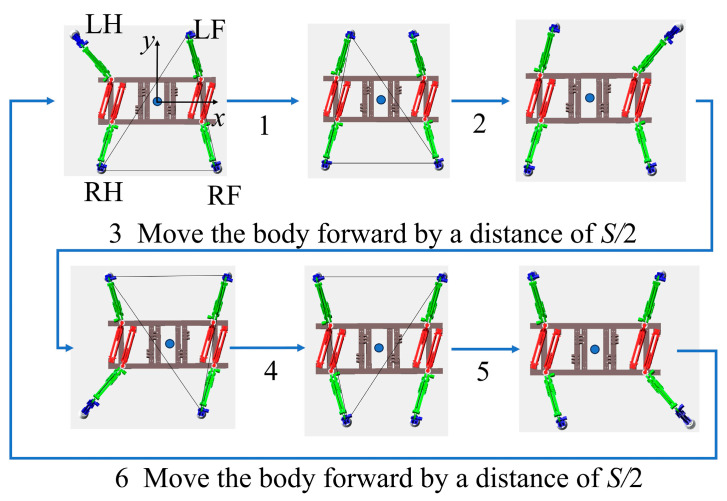
Crawling gait planning for a quadrupedal robot.

For quadrupedal robots, stability is maintained during crawling by ensuring that the center of gravity remains within the support polygon at all times. Design a crawling gait suitable for this structure by mimicking the crawling posture of the gecko. Gait planning is illustrated in Figure 8. The crawling gait features alternating limb movements with a low step frequency, enabling stable movement but limiting high-speed mobility. Diagonal gait involves synchronized movement of the opposite limbs, which is suitable for medium-to-high speeds. However, it is sensitive to disturbances, relies heavily on dynamic balance, and poses significant control challenges during underwater operation. Considering the characteristics of the application environment, selecting a crawling gait to ensure higher stability is a more practical solution.

*S* is the stride distance. The initial operating state of the module is when the pressures of the two hydraulic artificial muscles are balanced and their lengths are equal. When the pre-adjusted length of the steel wire rope is set to a certain fixed value and the two WHAMs of the joint module are in the same length position, the output torque and working range are at their maximum. The pose of the thigh module was horizontal to the ground, and the pose of the calf module was perpendicular to the ground. During robot crawling, Stage 1: Lift LH with step distance *S*. Based on quadruped stability criteria, assuming that the robot’s center of gravity aligns with the quadruped robot’s center of mass and remains within the triangle formed by the other three leg tips, stability is maintained after LH lift—the robot will not tip over. Then, in stage 2, the left front leg steps forward by distance *S*. Similarly, the robot’s center of gravity remains within the support polygon, preventing the quadruped robot from tipping over. Next, in stage 3, the body’s center of gravity was adjusted by translating the body forward by a distance *S*/2. At this point, all four leg joints simultaneously contact the ground, ensuring that the quadrupedal robot remains stable. Subsequently, the right hind leg and right front leg step forward sequentially, each with a stride length of *S*. Upon completing these steps, the robot enters Stage 6. The body’s center of gravity is then readjusted by translating forward by *S*/2 and returning to the initial position. This completes one cycle of the straight-line crawling gait. By repeating this gait planning process, the robot achieved a continuous forward crawling motion.

Owing to its small impact effect at the beginning and end of motion, the compound cycloidal wire is often used in trajectory planning of the foot end of robots. Therefore, in this study, an improved compound cycloidal wire was used as the trajectory planning curve for the leg lifting at the end of the foot. The support point coordinates of each leg are denoted as (*x_i_*, *y_i_*, *z_i_*), where *i* = 1, 2, 3, 4 correspond to the foot end center coordinates of LH, LF, RH, and RF, respectively. The mathematical expression of the swing phase is

(19) $$\left\{\begin{array}{l} x_{i}=iS(\frac{t}{T_{m}}-\frac{1}{2\pi }\sin(2\pi \frac{t}{T_{m}}))+mS,0\leq t\leq T_{m}\\ z_{i}=\left\{\begin{array}{c} 2H(\frac{t}{T_{m}}-\frac{1}{4\pi }\sin(4\pi \frac{t}{T_{m}}))+z_{0},0\leq t\leq \frac{T_{m}}{2}\\ 2H(1-\frac{t}{T_{m}}+\frac{1}{4\pi }\sin(4\pi \frac{t}{T_{m}}))+z_{0},\frac{T_{m}}{2}\leq t\leq T_{m} \end{array}\right. \end{array}\right.,$$

During motion of the LF, RF, RH and LH, *y_i_* = *y*_0_. *z*_0_ is the foot end initial height. *S* represents the step length. *H* denotes the leg lift height, and *T*_m_ is the one-leg lift cycle. *m* and *i* are stage-dependent parameters that vary across different phases. The swing phase parameters are listed in Table 4.

During the stage of the center of gravity, there is a shift in gait. Suppose that coordinates of each leg support point are (*x_i_*, *y_i_*, *z_i_*), where *i* = 1, 2, 3, 4 correspond to the foot end center coordinates of LH, LF, RH, and RF, respectively. The translation stage is the reverse process of the leg lifting swing, with a translation distance of *S*/2 and a translation period of *T*. Then, the formula for the translational trajectory at the end of each leg is

(20) $$\left\{\begin{array}{l} x_{i}=j\frac{S}{2}(\frac{T_{m}-t}{T_{m}}+\frac{1}{2\pi }\sin(2\pi \frac{t}{T_{m}}))+nS,\\ z_{i}=z_{0} \end{array}\right.$$

During motion of the LF, RF, RH and LH, *y_i_* = *y*_0_. *n* and *j* are stage-dependent parameters that vary across different phases. The values for the translation phase parameters are shown in Table 5.

**Figure 9:**
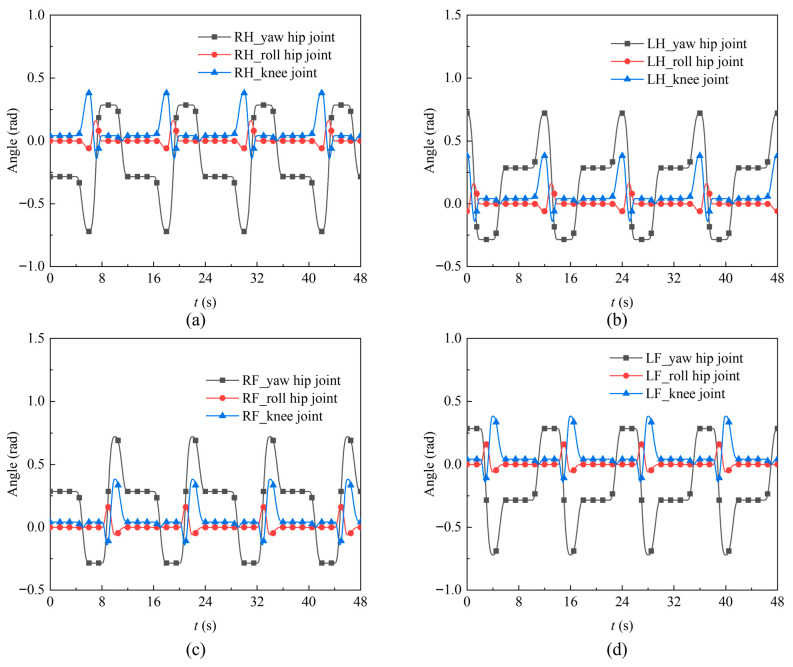
Variation curves of joint angles. (**a**) Angle variation curves of RH; (**b**) Angle variation curves of LH; (**c**) Angle variation curves of RF; (**d**) Angle variation curves of LF.

Based on the above formula, a Simulink program was developed for gait planning and solving the inverse kinematics of the quadruped robot’s leg joints. During the movement process, the module’s ideal working state is maintained near the pressure balance of the two WHAMs, with *H* = 100 mm, *S* = 800 mm, $y_{0}=l_{1}+l_{2}$ and $z_{0}=-l_{3}-161.71$. Using the parameters in Table 2, Table 3 and Table 4, the joint angle variation curves of each leg in the four gait planning cycle periods were obtained, as shown in Figure 9.

During the process of translation and leg lifting, under the influence of gravity, the end of the robot’s foot is subjected to a supporting force that varies over time. The magnitude of the supporting force affected the control effect of WHAM. More importantly, whether the leg joint actuated by WHAM can meet the actual requirements under the action of the entire machine’s gravity is a prerequisite for the robot’s crawling control. Therefore, it is necessary to conduct mechanical calculations for each moment of the crawling gait of the quadrupedal robot based on its parameters to determine the position with the greatest force.

### 3.4. The Determination of the Foot End Supporting Force

During the crawling process, the ends of the supporting legs were in contact with the ground. According to the static equilibrium calculation rule, three static equilibrium equations can be obtained as follows:

(1) Equilibrium of Forces (Vertical Direction)

(21) $$\sum_{i=1}^{4}G_{i}=m_{0}g,$$

(2) Regarding the torque balance of the *x*-axis

(22) $$\sum_{i=1}^{4}G_{i}y_{i}=0,$$

(3) Regarding the torque balance on the *y*-axis

(23) $$\sum_{i=1}^{4}G_{i}x_{i}=0,$$

During the swing phase, the foot end does not contact the ground, and thus, no supporting force is present. Three equilibrium equations can be used to solve the foot end supporting force on each of the remaining legs, and the solution results are unique. However, during the process of the center-of-gravity shift, because all four legs are in contact with the ground, there are four unknown supporting forces for the target to be solved, but only three independent solution equations. Therefore, in the process of the robot adjusting the center of gravity, there are infinitely many solutions for the supporting forces at the foot end of each leg. To obtain definite solutions, an additional limiting condition needs to be added when solving the foot end supporting force of the leg joint during the process of the center of gravity shift. Considering that the more load one leg joint bears, the less load the other leg joint bears on the same side. When the joint leg transitions from the swing phase to the supporting phase, the supporting force at the foot end instantly increases. To prevent instability of the robot caused by a drastic change in the supporting force, it is hoped that the supporting force of the leg joint can transition smoothly from the swing phase to the supporting phase. This can be achieved by actively controlling the pressure difference between the two WHAMs of the joint module. Therefore, to ensure a smooth change in the supporting force at the end of the foot during the translational phase of gait, the change in the supporting force at the end of one leg’s foot is defined as f=−Acos(πt/T)+A. *A* cosine curve is a common and simple function. Within half a cycle starting from time 0, a smooth transition of the supporting force amplitude from 0 to 2*A* can be achieved. Smoothly transitions the supporting force at the foot end of the leg joint from the swing phase to the supporting phase. The third stage of gait planning is expressed in matrix form as follows:

(24) $$\left[\begin{array}{c} m_{0}g\\ 0\\ 0\\ f \end{array}\right]=\left[\begin{array}{cccc} 1 & 1 & 1 & 1\\ y_{1} & y_{2} & y_{3} & y_{4}\\ x_{1} & x_{2} & x_{3} & x_{4}\\ 0 & 1 & 0 & 0 \end{array}\right]\left[\begin{array}{c} G_{1}\\ G_{2}\\ G_{3}\\ G_{4} \end{array}\right],$$

The solution matrix for the 6th stage of gait planning is given by:

(25) $$\left[\begin{array}{c} m_{0}g\\ 0\\ 0\\ f \end{array}\right]=\left[\begin{array}{cccc} 1 & 1 & 1 & 1\\ y_{1} & y_{2} & y_{3} & y_{4}\\ x_{1} & x_{2} & x_{3} & x_{4}\\ 0 & 0 & 0 & 1 \end{array}\right]\left[\begin{array}{c} G_{1}\\ G_{2}\\ G_{3}\\ G_{4} \end{array}\right],$$

**Figure 10:**
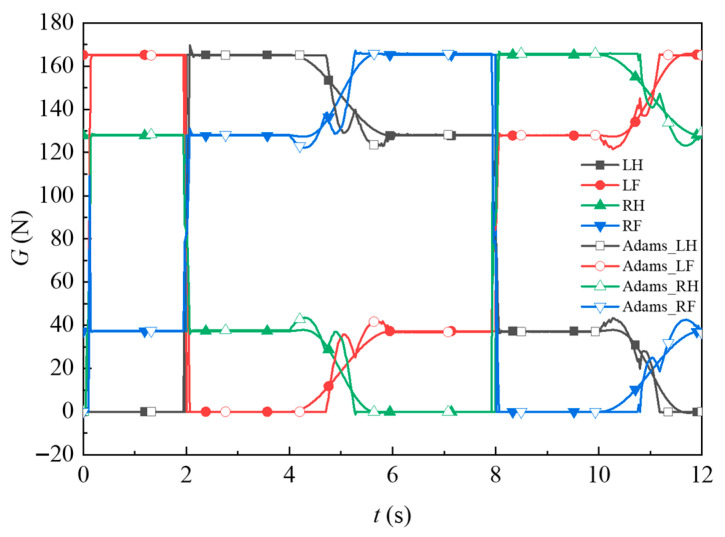
Changes in the supporting force at the end of the foot.

By substituting the parameters in Table 3, Table 4 and Table 5, the changes in the foot end supporting force of each leg during the center-of-gravity shift stage under the gait planning in Figure 8 are obtained, as shown in Figure 10.

To visually observe the load changes at the end of the foot, a combined simulation platform of MATLAB 2021b and dynamic simulation software was used to simulate the crawling gait of the quadrupedal robot, establish the contact force between the foot end and the ground, and establish a rotation pair at each rotating joint of the quadrupedal robot. The rotating joints were substituted into the angle curves of Figure 9 for the motion simulation. The simulation variation in the foot end supporting force for one gait cycle period was obtained, as shown in Figure 10.

The supporting force curve at the end of the quadruped robot’s leg shows that during each gait cycle, the force on each leg varies between a maximum of 165.17 N (16.85 kg) and a minimum of 0 N. In the swing phase, the theoretical calculations closely matched the results from the dynamic simulation, validating the accuracy of the theoretical formula for the leg-lifting phase. When a leg is in the swing phase, the joint of the other leg on the same side bears half the body load, as indicated by both the computational and simulation results. During the center-of-gravity translation phase, the dynamics simulation software only performs open-loop control of the joint angles for the quadrupedal robot model without controlling the joint torques. Based on collision contact theory, the simulation software provided a set of solutions. Fluctuations in the foot end supporting force can easily lead to unstable control. Therefore, in the theoretical calculations, a smoothing transition process was applied to the supporting force during the center-of-mass translation phase to avoid such fluctuations during this critical stage of movement.

## 4. Single-Leg Weight-Bearing Test

### 4.1. The Relationship Between the Pressure Difference of WHAMs and the Load

**Figure 11:**
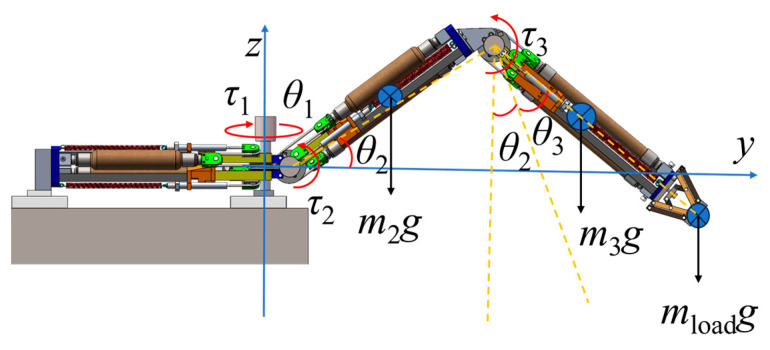
Force analysis of the single leg under load.

In practical applications, the root module is fixed to the frame and the load from the fuselage acts on the root module. However, it is difficult to simulate this loading condition directly using a single-leg prototype. The analysis showed that the calculated supporting force at the foot end of a single leg is perpendicular to and upward from the ground. By adding weights at the foot end, a force in the opposite direction can be simulated, the only difference being the direction, thus indirectly testing the load-bearing capacity of a single leg. If a single leg can lift a weight equivalent to the load, then by switching the WHAMs, it can also bear a distributed load of the same magnitude as the fuselage. Therefore, in the experiment, the load-bearing capacity was tested by adding weights at the foot end while the single leg was fixed to a platform, as shown in Figure 11.

To reduce computational complexity, the mass of the joint module was simplified to the center of the joint module. $m_{\mathrm{load}}$ is the mass of the additional external load. $\tau_{1}$ represents the torque due to gravity on the yaw hip joint, $\tau_{2}$ represents the torque due to gravity on the roll hip joint, and $\tau_{3}$ represents the torque due to gravity on the knee joint. Based on the static equilibrium relationship, the theoretical relationship between the output torque of each joint wheel and load is derived as follows:

(26) $$\tau_{1}=0,$$

(27) $$\tau_{2}=m_{3}g[l_{2}\cos\theta_{2}+\frac{a_{3}}{2}\sin(\theta_{3}+\theta_{2})]+m_{2}g\frac{l_{2}}{2}\cos\theta_{2}+m_{\mathrm{load}}g[l_{2}\cos\theta_{2}+a_{3}\sin(\theta_{3}+\theta_{2})],$$

(28) $$\tau_{3}=m_{3}g\frac{a_{3}}{2}\sin(\theta_{3}+\theta_{2})+m_{\mathrm{load}}ga_{3}\sin(\theta_{3}+\theta_{2}),$$

$a_{3}$ denotes the distance from the external load to the knee joint during the experiment. Equation (10) establishes the relationship between the pressure difference of WHAMs and the module torque. Substituting Equations (26)–(28) into Equation (10) yields the pressure difference–load model of the leg joints as follows:

(29) $$\Delta p_{1}=\frac{k_{2}p_{0}R\theta_{1}}{k_{1}+k_{3}{\left(R\theta_{1}\right)}^{2}},$$

(30) $$\Delta p_{2}=\frac{k_{2}p_{0}R\theta_{2}}{k_{1}+k_{3}R^{2}{\theta_{2}}^{2}}+\frac{m_{2}gl_{2}\cos\theta_{2}}{\left(k_{1}+k_{3}R^{2}{\theta_{2}}^{2}\right)R}+\frac{2m_{3}g[l_{2}\cos\theta_{2}+\frac{a_{3}}{2}\sin(\theta_{2}+\theta_{3})]}{\left(k_{1}+k_{3}R^{2}{\theta_{2}}^{2}\right)R}+\frac{2m_{\mathrm{load}}g[l_{2}\cos\theta_{2}+a_{3}\sin(\theta_{2}+\theta_{3})]}{\left(k_{1}+k_{3}R^{2}{\theta_{2}}^{2}\right)R},$$

(31) $$\Delta p_{3}=\frac{k_{2}p_{0}R\theta_{3}}{k_{1}+k_{3}R^{2}{\theta_{3}}^{2}}+\frac{m_{3}ga_{3}\sin(\theta_{2}+\theta_{3})}{\left(k_{1}+k_{3}R^{2}{\theta_{3}}^{2}\right)R}+\frac{2m_{\mathrm{load}}ga_{3}\sin(\theta_{2}+\theta_{3})}{\left(k_{1}+k_{3}R^{2}{\theta_{3}}^{2}\right)R},$$

### 4.2. Introduction to the Single-Leg Test Bench

**Figure 12:**
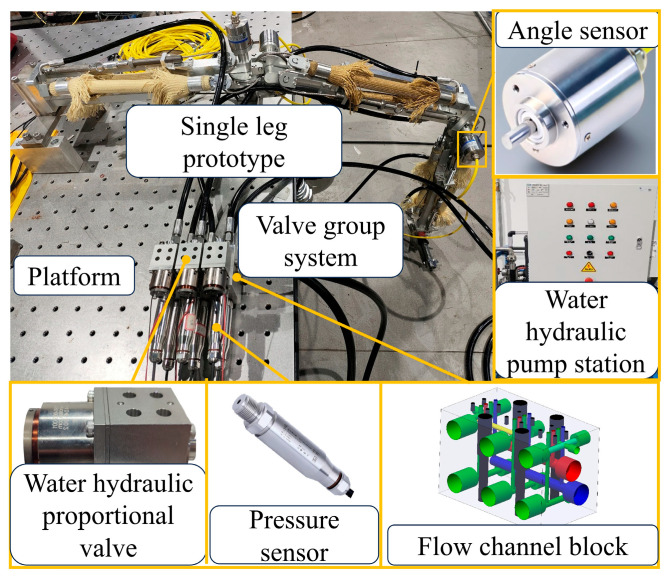
Single-leg test bench.

A single-leg prototype was fabricated and manufactured, and the corresponding experimental testbed was constructed, as shown in Figure 12. The single-leg test bench includes a water hydraulic pump station, single-leg prototype, valve group system and platform. The valve group system consisted of three water hydraulic proportional valves and a flow channel block. The single-leg prototype was securely bolted to the platform and was equipped with angle sensors at its three joints to measure the joint angles in real time. Each joint module adjusts the pressure of the two WHAMs through a water hydraulic proportional valve. The valve core of the water hydraulic proportional valve is fixed to the voice coil motor. Controlling the opening degree of the valve port can achieve pressure control of the WHAM. Three water hydraulic proportional valves were integrated into the flow channel block to facilitate installation and commissioning. In addition, the flow channel block was equipped with pressure sensors to monitor the internal pressure changes in WHAM in real time. The high-pressure water in the system was provided through a water hydraulic pump station.

### 4.3. Test and Analysis of Static Ultimate Load Capacity of a Single Leg

**Figure 13:**
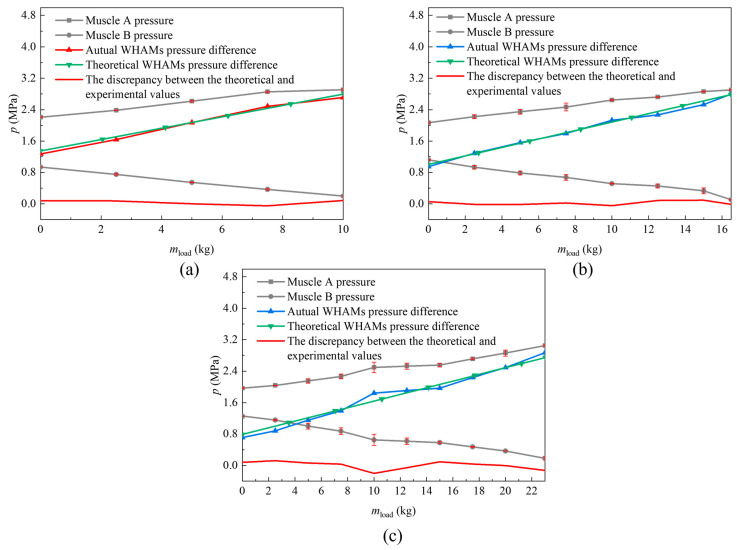
Relationship between WHAMs pressure and load in the thigh module. (**a**) Muscle pressure-load curve of the thigh module at a pre-adjusted wire rope length of 90 mm; (**b**) Muscle pressure-load curve of the thigh module at a pre-adjusted wire rope length of 60 mm; (**c**) Muscle pressure-load curve of the thigh module at a pre-adjusted wire rope length of 30 mm.

To evaluate the load-bearing performance of a single leg under practical conditions, static testing was performed on a single-leg prototype. The pre-adjustment lengths of the steel wire rope in the thigh module were set to 30, 60, and 90 mm. During testing, the internal pressure of the water hydraulic artificial muscle was adjusted to ensure that the thigh and lower leg modules remained in a balanced position with a joint angle of 0°, at which point the joint module could output its maximum torque. Loads were sequentially applied to the end of the single-leg prototype, starting from 0 kg and incrementally increasing to 2.5 kg, 5.0 kg, 7.5 kg, 10.0 kg, 12.5 kg, 15.0 kg, 17.5 kg, 20.0 kg, and 23.0 kg. The final test results revealed the relationship between the internal pressure of WHAM and the applied load, as shown in Figure 13.

During the tests, the pump pressure was set to 3 MPa. Owing to the backpressure, the maximum adjustable pressure was limited to 2.8 MPa. The results showed that, as the load increased, a greater pressure difference between the two WHAMs in the thigh module was required. Under the same load, the theoretical pressure difference across the WHAMs was approximately equal to the actual value. When the pre-adjusted length of the steel wire ropes on both sides of the thigh module was set to 90 mm, the maximum deviation between the theoretical and actual pressure differences under identical loads was 0.08 MPa. The maximum load capacity measured during the tests was 10.00 kg, whereas the theoretical capacity was 10.03 kg. When the pre-adjusted length was reduced to 60 mm, the maximum deviation between the theoretical and actual pressure differences increased to 0.09 MPa under the same load conditions. The experimental maximum load capacity was 16.50 kg, compared to the theoretical value of 16.64 kg. Further reduction of the pre-adjusted length to 30 mm resulted in a maximum pressure difference deviation of 0.20 MPa. Under this setting, the experimental maximum load capacity reached 23.00 kg, with a theoretical value of 23.69 kg. Based on the calculated supporting force, this configuration satisfied the load requirements of the fuselage. With the pre-adjusted length set to 30 mm, the initial contraction ratio of the WHAMs was 0.05. According to the relationship between the joint wheel rotation angle and the change in muscle length $\theta_{\max}=2l_{0}\varepsilon_{0}/R$, the maximum rotation range of the joint wheel was ±0.75 rad. Based on the roll hip joint angle curve from the gait planning, the maximum required rotation angle was 0.16 rad. Therefore, at a pre-adjusted length of 30 mm, the leg joint satisfied both the load capacity and rotation range requirements.

The tests demonstrate that adjusting the pre-adjusted length of the steel wire ropes enables the modulation of the load-bearing capacity of the leg joint. A smaller pre-adjusted length resulted in a higher load capacity. When the pre-adjusted length was 0 mm, the joint reached its maximum load-bearing capacity but lost its ability to move. In practical applications, the robot’s load on the robot should first be evaluated. The pre-adjusted lengths of the steel wire ropes can then be calibrated using theoretical formulas to ensure sufficient load capacity while retaining an adequate range of motion to meet the rotation requirements specified by the gait plan.

### 4.4. Dynamic Loaded Motion Test and Analysis of Single Leg

Static tests were conducted with the thigh module deflection angle set to 0°, with the aim of evaluating the maximum load-bearing capacity of the leg. However, these tests did not analyze leg motion performance. To assess the leg’s ability to perform loaded lifting movements, dynamic tests were performed out with the initial contraction rate of the thigh joint module set to 0.05, and those of the root and calf modules set to 0.15.

**Figure 14:**
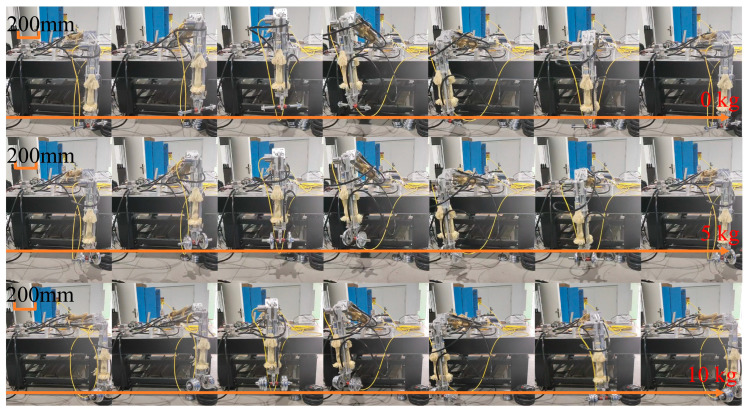
Motion diagram of the single leg under different loads.

Load-bearing trials were performed during the leg lifting and horizontal moving phases. The joint motion of the robotic leg during lifting follows a modified cycloidal trajectory. During the tests, the parameters in Equation (19) were set as *i* = 1 and *m* = −1/2, with lifting height *H* = 200 mm and step length *S* = 800 mm. In Equation (20), *j* = 2 and *n* = −1/2 were used. The motion and translation cycles were set to 25 s for the experiment. Loads were applied incrementally at the end of the leg, with masses increasing from 0 to 5 kg and then to 10 kg. The motion of the leg joints during the tests was recorded, as shown in Figure 14.

**Figure 15:**
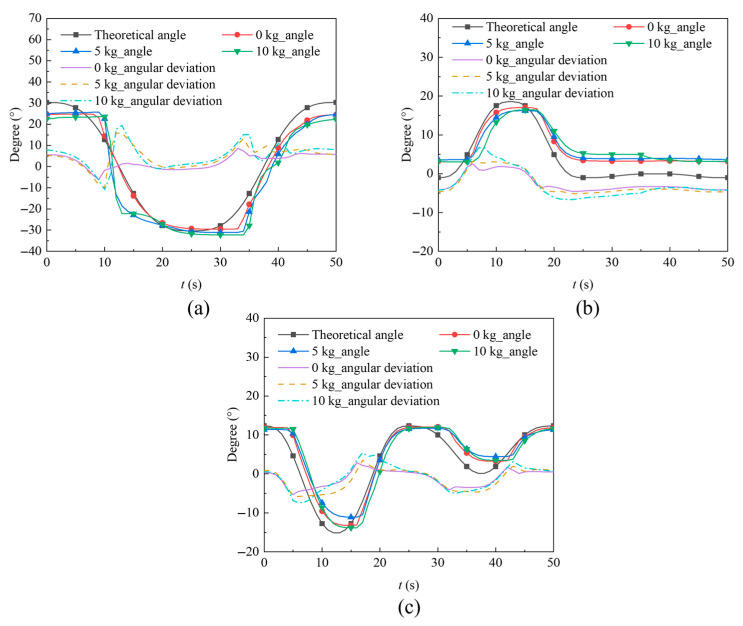
Angle-tracking performance. (**a**) Angle tracking curves of the yaw hip joint under different loads; (**b**) Angle tracking curves of the roll hip joint under different loads; (**c**) Angle tracking curves of the knee joint under different loads.

During the experiments, closed-loop PID control was implemented based on the joint angle feedback. The resulting angle-tracking performance is shown in Figure 15. The Ziegler-Nichols critical proportional method [34] was adopted for PID parameter tuning. When tuning any single degree of freedom, the other two degrees of freedom remained fixed. The proportional gain *k*_P_ was gradually increased from zero until the system exhibited sustained oscillations. Based on the principles of the Ziegler-Nichols method, the PID parameters for the three joint modules were obtained as shown in Table 6.

**Figure 16:**
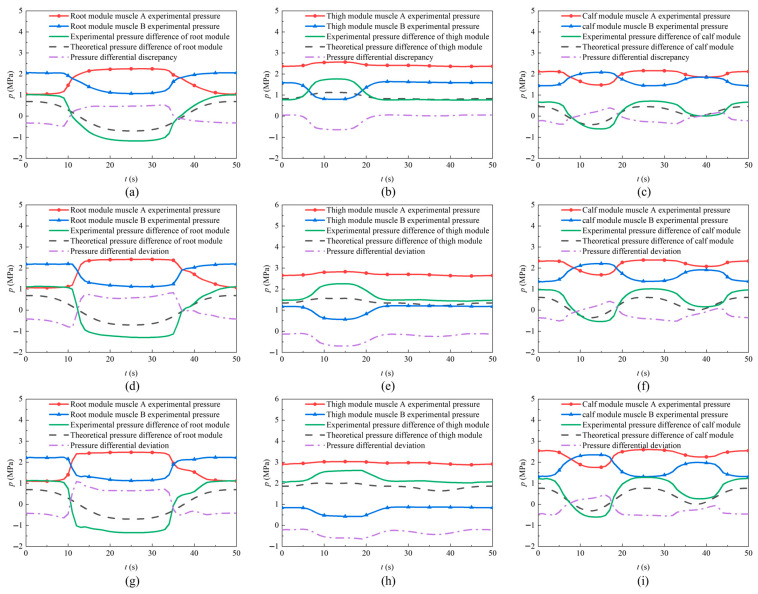
The variations in WHAMs pressure during motion under loads of 0 kg, 5 kg, and 10 kg for each joint module. (**a**) Pressure difference in the root module under no-load conditions; (**b**) Pressure difference in the thigh module under no-load conditions; (**c**) Pressure difference in the calf module under no-load conditions; (**d**) Pressure difference in the root module under a 5 kg load; (**e**) Pressure difference in the thigh module under a 5 kg load; (**f**) Pressure difference in the calf module under a 5 kg load; (**g**) Pressure difference in the root module under a 10 kg load; (**h**) Pressure difference in the thigh module under a 10 kg load; (**i**) Pressure difference in the calf module under a 10 kg load.

The dynamic test was conducted on a LabVIEW measurement and control platform. The LabVIEW platform includes LabVIEW 2021, an NI-9264 analog voltage output module, an NI-9205 data acquisition module, and an ATA300 power amplifier. A control program was programmed in the LabVIEW software, which outputs control signals via the analog voltage output module to the ATA300 power amplifier to control the valve system, thereby achieving drive control of the leg joint. The sampling rate set in the LabVIEW software is 2000 Hz, and the number of samples to read is 50. The variations in the pressure within the WHAMs of each joint module under these conditions are shown in Figure 16. Owing to the inherent flexibility of the WHAMs and their high sensitivity to load variations, the control accuracy became more challenging to maintain as the load increased.

Under no-load conditions, the maximum absolute angular deviation of the yaw hip joint’s tracking trajectory measured 6°. With a 5-kg load applied, this deviation increased to 15.6°, and further reached 19.5° under 10-kg loading. As the load intensified, the tracking accuracy of the yaw hip joint progressively decreased. In the angle-tracking curve of the yaw hip joint, the largest deviation occurred within the time interval of 10 s to 15 s, which corresponds to the swing phase of the leg. During this phase, the leg undergoes large angular displacements, and significant motion of the center of mass introduces substantial inertial effects that adversely affect the control precision.

Under no-load conditions, the maximum absolute angular deviation of the roll hip joint’s tracking trajectory measured 4.6°. With a 5-kg load applied, this deviation increased to 5.2°, and further reached 6.7° under 10-kg loading. As the load increased, the roll hip joint also exhibited a gradual rise in the absolute angular deviation of its tracking trajectory, though with a more limited magnitude of increase, demonstrating comparatively stronger robustness.

Under no-load conditions, the maximum absolute angular deviation of the knee joint’s tracking trajectory measured 5.3°. With a 5-kg load applied, this deviation increased to 5.8°, and further reached 7.4° under 10-kg loading, demonstrating comparatively stronger robustness. The knee joint and roll hip joint exhibited good tracking performance between the experimental and desired angles under all the three loading conditions. This can be attributed to the relatively small swing amplitude of the joint module during motion, resulting in minimal influence from changes of the load. Consequently, the control accuracy is less affected.

Under no-load conditions, the maximum deviation between the theoretical and experimental pressure differences in the root module is 0.53 MPa. When the load was increased to 5 kg and 10 kg, the maximum deviations rose to 0.83 MPa and 1.08 MPa, respectively. The deviation between the theoretical and experimental pressure differences increased with load. This trend can be attributed to two main factors: first, the increased load resulted in greater motion inertia, which reduced the control accuracy of the yaw hip joint; second, the elevated load led to increased internal friction within the joint module, necessitating a larger pressure difference in the WHAMs for actuation.

Under no-load conditions, the maximum deviation between the theoretical and experimental pressure differences in the thigh module is 0.53 MPa. When the load was increased to 5 kg and 10 kg, the maximum deviations rose to 0.70 MPa and 0.65 MPa, respectively. It was observed that under low-load conditions, the magnitude of pressure decrease in Muscle B was significantly greater than the magnitude of pressure increase in Muscle A in the thigh module, whereas the magnitudes of pressure variation in both muscles A and B were approximately equal in the root and calf modules. The theoretical analysis employed a simplified approach in which Δp/2 was used to represent both the magnitude of the pressure increase in Muscle A magnitude of the pressure decrease in Muscle B. This simplification resulted in a good agreement between the theoretical calculations and experimental data when the joint module operated near the pressure equilibrium point. At a 10 kg load, the difference between the descent magnitude in Muscle B and the ascent magnitude in Muscle A became smaller, leading to improved fidelity of the theoretical pressure difference model to the experimental data.

For the calf module, the deviation between theoretical and experimental pressure differences increased with load. Under no-load conditions, the maximum deviation was 0.40 MPa; it rose to 0.51 MPa under a 5 kg load and reached 0.56 MPa under a 10-kg load. This phenomenon is primarily owing to the influence of nonlinear friction within the joint module.

As the end load gradually increased, the maximum pressure in the muscles of each module also increased. The thigh module exhibited greater sensitivity to load variations with more pronounced pressure changes under increasing loads. When the end load reached 10 kg, the pressure in Muscle A of the thigh module approached the system’s maximum pressure of 3 MPa during the leg-lifting motion, indicating that the system had reached its load limit. Thus, the output torque of the thigh module constrains the load-bearing capacity of the single leg.

The increase in experimental pressure in the muscles of the root module was primarily attributed to the increased friction torque. As the load increased, the friction in the yaw hip joint increased, leading to a greater pressure difference between the two WHAMs in the root module.

In the dynamic tests, the theoretical and experimental pressure differences were compared. Owing to the presence of dry friction in multiple internal structures of the joint module, the friction force varies with the applied load, making it challenging to accurately model friction using mathematical expressions. The experiments were performed only on one leg of the robot, not on the entire system. The interactions between the legs and the overall behavior of the robot have not been practically verified.

WHAMs represent a type of soft actuation module that requires sufficient flow supply during pressure regulation. The processes of water intake and discharge require finite time, making it challenging to simultaneously ensure both rapid response and precise control in motion execution, thereby limiting their application in scenarios demanding high efficiency. The load-bearing capacity of the leg joints is correlated with the pressure in the WHAMs. To ensure operational safety, the system pressure was set to 3 MPa, which somewhat constrains the maximum load capacity of the leg. The current pressure difference–load model only accounts for gravitational effects, while frictional forces within the joint that vary with load were not accounted for. Future work will focus on incorporating these factors to enhance the adaptability and accuracy of the load model.

The advantages of WHAMs are particularly evident during operation in aquatic environments. However, the technology is currently still in the preliminary terrestrial testing phase. Future iterations are expected to enable genuine application of WHAMs in underwater robotics, where factors such as buoyancy, hydrodynamic drag, and other aquatic influences will be systematically incorporated into the robotic design and control strategies during operational deployment.

## 5. Conclusions

In this study, the load-bearing capacity of the leg joints of a quadruped robot actuated by WHAMs was determined and the following conclusions were drawn:

(1) A novel WHAM-actuated quadrupedal robot structure was proposed. The WHAMs were applied to the field of quadrupedal robots.
(2) A theoretical model correlating the pressure difference in the WHAMs of the leg joint module and end load was developed. Static tests verified that the deviation between the theoretical and experimental pressure differences in the thigh module under the same load was within an acceptable range, whereas the dynamic tests also demonstrated good agreement with the theoretical predictions. These results provide a theoretical basis for setting the pre-adjusted length of the steel wire ropes in WHAM modules.
(3) Static tests showed that at an initial contraction rate of 0.05 of the WHAMs, the single leg could bear a maximum load of 23.00 kg, exceeding the no-load requirement of 16.85 kg by a redundancy of 6.15 kg. In the dynamic tests, the leg could perform lifting motions with a load of 10 kg.

The quadruped robot actuated by WHAMs has a good load capacity. The load capacity of the leg joints was adjustable, and the load output could be adjusted according to different working conditions. The pressure difference–load model of the leg joints provides a theoretical basis for adjusting the load capacity of the leg joints.

## Figures and Tables

**Table 1:** Parameters of the joint module.

Parameters	Units	Specification
Initial length of WHAM, $l_{0}$	mm	300
Initial radius of WHAM, $r_{0}$	mm	15
Stress adjustment parameter, $\kappa_{F}$		0.73
Strain adjustment parameter, $\kappa_{\varepsilon }$		1.13
Joint wheel radius, *R*	mm	40
Initial contraction rate of WHAM, $\varepsilon_{0}$		0~0.3
Initial weaving angle, $\alpha_{0}$	°	25
Initial pressure, $p_{0}$	MPa	$(p_{1}+p_{2})/2$

**Table 2:** Average morphological measurements from a gecko [33].

Species	Snout Vent Length	Upper Limb	Lower Limb
Rhoptropus boultoni	41.8 ± 1.6 mm	13.3 ± 0.5 mm	13.0 ± 0.5 mm

**Table 3:** Parameters of quadrupedal robot.

Parameters	Units	Specification
Half the distance between the yaw hip joints along the *x*-axis, *l*	mm	685.0
Half the distance between the yaw hip joints along the *y*-axis, *ω*	mm	322.0
Distance between the yaw hip joint and the roll hip joint, $l_{1}$	mm	60.0
The distance between the roll hip joint and the knee joint, $l_{2}$	mm	623.6
The distance from the knee joint to the foot, $l_{3}$	mm	555.5
Body mass (including root modules), $m_{0}$	kg	33.7
Thigh module mass, $m_{2}$	kg	5.5
Calf module mass, $m_{3}$	kg	6.5
Yaw hip joint rotation angle, $\theta_{1}$	degree	−75~75
Roll hip joint rotation angle, $\theta_{2}$	degree	−90~90
Knee joint rotation angle, $\theta_{3}$	degree	−90~90

**Table 4:** Swing Phase Parameters.

Schemes	Legs	*m*	*i*
1	LH	−3/4	1
2	LF	−1/4	1
4	RH	3/4	−1
5	RF	1/4	−1

**Table 5:** Translation phase parameters.

Stages	Legs	*n*	*j*
3	LH	−1/4	1
	LF	1/4	1
	RH	3/4	−1
	RF	1/4	−1
6	LH	−3/4	1
	LF	−1/4	1
	RH	1/4	−1
	RF	−1/4	−1

**Table 6:** PID parameters.

Target	*k*_P_	*k*_I_	*k*_D_
Root module	0.4	0.3	0.075
Thigh module	0.8	0.6	0.14
Calf module	0.6	0.3	0.078

## Data Availability

Data are contained within the article.
